# Oxidation of HMGB1 Is a Dynamically Regulated Process in Physiological and Pathological Conditions

**DOI:** 10.3389/fimmu.2020.01122

**Published:** 2020-06-24

**Authors:** Michele Ferrara, Ginevra Chialli, Lorena Maria Ferreira, Elena Ruggieri, Giorgia Careccia, Alessandro Preti, Rosanna Piccirillo, Marco Emilio Bianchi, Giovanni Sitia, Emilie Venereau

**Affiliations:** ^1^Division of Genetics and Cell Biology, Chromatin Dynamics Unit, IRCCS San Raffaele Scientific Institute, Milan, Italy; ^2^Vita-Salute San Raffaele University, Milan, Italy; ^3^Division of Immunology, Transplantation and Infectious Diseases, Experimental Hepatology Unit, IRCCS San Raffaele Scientific Institute, Milan, Italy; ^4^HMGBiotech srl, Milan, Italy; ^5^Department of Neurosciences, Mario Negri Institute for Pharmacological Research IRCCS, Milan, Italy

**Keywords:** inflammation, regeneration, injury, leukocyte, tumor, cancer cachexia, muscle, liver

## Abstract

Acute inflammation is a complex biological response of tissues to harmful stimuli, such as pathogens or cell damage, and is essential for immune defense and proper healing. However, unresolved inflammation can lead to chronic disorders, including cancer and fibrosis. The High Mobility Group Box 1 (HMGB1) protein is a Damage-Associated Molecular Pattern (DAMP) molecule that orchestrates key events in inflammation by switching among mutually exclusive redox states. Fully reduced HMGB1 (frHMGB1) supports immune cell recruitment and tissue regeneration, while the isoform containing a disulphide bond (dsHMGB1) promotes secretion of inflammatory mediators by immune cells. Although it has been suggested that the tissue itself determines the redox state of the extracellular space and of released HMGB1, the dynamics of HMGB1 oxidation in health and disease are unknown. In the present work, we analyzed the expression of HMGB1 redox isoforms in different inflammatory conditions in skeletal muscle, from acute injury to muscle wasting, in tumor microenvironment, in spleen, and in liver after drug intoxication. Our results reveal that the redox modulation of HMGB1 is tissue-specific, with high expression of dsHMGB1 in normal spleen and liver and very low in muscle, where it appears after acute damage. Similarly, dsHMGB1 is highly expressed in the tumor microenvironment while it is absent in cachectic muscles from the same tumor-bearing mice. These findings emphasize the accurate and dynamic regulation of HMGB1 redox state, with the presence of dsHMGB1 tightly associated with leukocyte infiltration. Accordingly, we identified circulating, infiltrating, and resident leukocytes as reservoirs and transporters of dsHMGB1 in tissue and tumor microenvironment, demonstrating that the redox state of HMGB1 is controlled at both tissue and cell levels. Overall, our data point out that HMGB1 oxidation is a timely and spatially regulated process in physiological and pathological conditions. This precise modulation might play key roles to finetune inflammatory and regenerative processes.

## Introduction

Inflammation is commonly perceived as a detrimental process and people often react to its five signs (pain, heat, redness, swelling, and loss of function) by taking anti-inflammatory drugs. Indeed, many chronic and degenerative diseases are associated to inflammatory processes, but inflammation is also important for the elimination of infections, the clearance of damaged cells and the regeneration of tissue (1). Many of the mechanisms that link inflammation to damage repair and regeneration in mammals are conserved during evolution, underlying the importance of this physiological process. Hence, chronic unresolved inflammation can lead to tissue damage and chronic disorders, including cancer and fibrosis, but self-limiting acute inflammation is essential for a proper healing process.

The Damage-Associated Molecular patterns (DAMPs) have been identified as key mediators of inflammation in response to infection or tissue damage (2). These sophisticated molecules have physiological roles inside the cell and, without damage, they are hidden to the immune system. Upon injury, DAMPs are exposed to the extracellular environment where they acquire additional functions: they alert the body about danger and contribute to inflammatory response and tissue repair (3). The High Mobility Group Box 1 protein fits all the criteria of DAMPs: it leads a double life having both intracellular and extracellular functions. HMGB1 has been first identified as a nuclear non-histone protein that regulates many processes in the nucleus from DNA repair to nucleosome dynamics (4, 5). However, HMGB1 is a very motile protein that can translocate to the cytoplasm and be passively released following traumatic death or actively secreted during severe stress to alert other cells of danger (6). This DAMP has been characterized as an inflammatory mediator, inducing both leukocytes recruitment and production of inflammatory cytokines and chemokines (7–9). Interestingly, the activities of HMGB1 in the extracellular microenvironment are tightly regulated by its redox state (7, 10, 11).

The HMGB1 protein is composed of two DNA-binding domains, called A box and B box, and of an acidic tail. This redox-sensitive protein contains 3 cysteines: C23 and C45 in the A box, which can form a disulphide bond, and the unpaired C106 in the B box. Notably, the redox state of these cysteines modulates the extracellular activities of HMGB1 and dictates its binding to different receptors. Fully reduced HMGB1 (frHMGB1) associates with the chemokine CXCL12 and activates the CXCR4 receptor, which recruits circulating leukocytes and stem cells to the site of damage, promoting tissue regeneration (7, 12, 13). Conversely, HMGB1 containing a disulphide bond (dsHMGB1) induces the expression of pro-inflammatory cytokines and chemokines by macrophages through its binding to MD-2, the TLR4 adaptor, or to the Receptor for Advanced Glycation End products (RAGE) (13, 14). Further cysteine oxidation to sulfonates by reactive oxygen species (ROS) abrogates both activities (7). It has been reported that HMGB1 inside the nucleus is fully reduced in normal conditions (15). It has also been suggested that the tissue itself determines the redox state of the extracellular space and most probably of released HMGB1, although it has not been demonstrated yet.

In the present work, we analyzed the expression of HMGB1 redox isoforms in different inflammatory conditions in skeletal muscle, from acute injury to chronic conditions of muscle wasting such as cancer cachexia, in tumor microenvironment, in spleen, and in liver after drug intoxication. Interestingly, we found that the presence of dsHMGB1 was tightly associated with an inflammatory state and we identified leukocytes as a main source of dsHMGB1. Overall, our data point at dsHMGB1 as a biomarker of inflammation and as a therapeutic target to dampen the inflammatory response.

## Materials and Methods

### Mice and Models

Eight-wk-old C57BL/6 and Balb/c WT mice were purchased from Charles River Laboratories. C57BL/6 HMGB1 fl/fl and HMGB1 fl/fl:MyoD-Cre (thereafter termed mKO) mice were bred in the animal facility at San Raffaele Scientific Institute. In mKO mice, Cre recombinase is under the control of MyoD promoter to knock out HMGB1 in all myogenic cells. All mice were housed under standard or specific pathogen–free conditions and allowed access to food and water *ad libitum* with the exception of the 16 h fasting prior to acetaminophen (APAP) injection, as described below. All experimental protocols were approved by the San Raffaele Institutional Animal Care and Use Committee (IACUC 838, 972, and 1,111) in accordance with Italian law. All efforts were made to minimize suffering.

In the acute muscle injury model, animals were anesthetized by intraperitoneal injection of Avertin (T48402, 2,2,2-Tribromoethanol 97%; Sigma-Aldrich), and sterile injury was induced by injection of 50 μl of 15-μM cardiotoxin (CTX, C9759 Sigma-Aldrich) in tibialis anterior or triceps muscles. WT mice were euthanized at 1 h, 6 h, 1 d, 3 d, 5 d or 7 d after CTX injection, while HMGB1 mKO mice were euthanized 1, 2, 5, and 7 d after CTX injection. Muscles were collected and either sectioned for histological analyses or subjected to protein quantification for Western blot analyses.

For the liver acute injury, 8-wk-old C57BL/6 males were fasted 16 h before intraperitoneal injection of 300 mg/Kg (body weight) APAP (Sigma-Aldrich) dissolved in sterile warm saline. Mice were i.p. injected with APAP or control saline, and after 1, 2, 3 and 7 days, were i.v. injected with 5 mg/Kg (body weight) of Evans Blue (Sigma-Aldrich) followed by euthanasia 30 min later. Spleen and liver were collected either for histological analyses or subjected to protein quantification by Western blot analyses. At the indicated time points, blood was collected for serum Alanine Aminotransferase (sALT) and HMGB1 quantifications.

For the cancer cachexia models, Lewis lung carcinoma (LLC) cells and C26 colon adenocarcinoma cells were maintained in DMEM (ThermoFisher) with 10% Fetal Bovine Serum (FBS). C57BL/6 and Balb/c WT mice were subcutaneously injected on the right flank with 5 × 10^6^ LLC cells or 1 × 10^6^ C26 cells, respectively, in 100 μl of Phosphate Buffered Saline (PBS). Blood was collected for HMGB1 quantification just before euthanasia. LLC- and C26-bearing mice were sacrificed 3- and 2-weeks post-injection of cancer cells, respectively. Skeletal muscles (tibialis anterior, quadriceps, gastrocnemius) and tumors were collected and either sectioned for histological analyses or subjected to protein quantification by Western blot analyses.

### Histology and Immunohistochemistry

Tibialis anterior muscles were fixed with 4% buffered paraformaldehyde solution for 3 h, then dehydrated in 15 and 30% sucrose and subsequently frozen in liquid nitrogen–cooled isopentane. Serial muscle sections, 8-μm thick, were then stained with anti-HMGB1 (1:800, ab18256 Abcam) and anti-CD45 (1:1000, ab10558 Abcam) antibodies.

Livers were collected and pieces of liver were either fixed with 4% buffered paraformaldehyde solution or zinc-formalin. The livers fixed over-night with 4% buffered paraformaldehyde solution were then equilibrated in 10, 20, and 30% sucrose, embedded in OCT for quick freezing at −80°C and cryosectioned (20 μm thickness) for subsequent fluorescent detection of Evans Blue damaged areas. The liver samples fixed in zinc-formalin were then embedded in paraffin, cut and stained with hematoxylin/eosin, anti-CD45 (1:1000, ab10558 Abcam), or anti-HMGB1 (1:800, ab18256 Abcam) antibodies.

Tumors were collected and fixed 24 h in formalin and then transferred in 70% ethanol solution. Fixed tumors were then embedded in paraffin, cut, and stained with anti-HMGB1 (1:800, ab18256 Abcam) and anti-CD45 (1:1000, ab10558 Abcam) antibodies.

### Image Acquisition and Analyses

Bright-field images were taken with a Leica DM750 microscope equipped with Leica ICC50 HD camera or with a Zeiss AxioImager M2m with AxioCam MRc5. Representative images were acquired at 20x magnification and analyzed by using ImageJ software (http://rsbweb.nih.gov/ij/).

Confocal images were acquired using a Leica TCS SP5 confocal system (Leica Microsystems) available at the SRSI Advanced Light and Electron Microscopy BioImaging Center (ALEMBIC). Twenty-micrometer z-stacks were projected in 2D and processed using Imaris image processing software.

### Leukocyte Isolation and Lysates

Peripheral blood mononuclear cells (PBMCs) were isolated from buffy coats of human healthy donors by Ficoll-Paque density centrifugation as previously described (7). PBMCs were then plated in RPMI 10% FBS and lysed after 30 min or treated with either combination of 1 μg/ml anti-CD3 (16-0037-85, Life Technologies) and 1 μg/ml anti-CD28 (16-0289-85, Life Technologies) or 1 μg/ml LPS (L4641, Sigma-Aldrich), and lysed after 24 or 72 h.

Intrahepatic leukocytes (IHLs) isolation was performed as previously described (16). Both PBMCs and IHLs (2–10 × 10^6^ cells) were lysed in 100 μl of RIPA buffer (50 mM Tris-HCl-pH 7.4, 1% IGEPAL, 0.5% Na-deoxycholate, 0.1% SDS, 150 mM NaCl, 2 mM EDTA, 50 mM NaF).

### Supernatant Collection and Tissue/Cell Lysates

Isolated muscles were incubated overnight at 4°C in 200 μl of Phosphate Buffered Saline with protease inhibitors cocktail (P8340, Sigma-Aldrich). Supernatants were collected and centrifuged at 12,000 rpm for 15 min at 4°C. Pellet was discarded and supernatants were analyzed by Western blot assays.

Tissues (muscles, tumor masses, livers, spleens) and cells (PBMCs, IHLs, tumor cells) were lysed in RIPA buffer (50 mM Tris-HCl-pH 7.4, 1% IGEPAL, 0.5% Na-deoxycholate, 0.1% SDS, 150 mM NaCl, 2 mM EDTA, 50 mM NaF) with protease inhibitors cocktail. Tissues were disrupted in RIPA buffer with TissueLyser LT (Qiagen). Lysates were then centrifuged at 12,000 rpm for 15 min at 4°C and the supernatants were collected.

### Western Blot Assays

Total protein content in muscle, tumor, liver, spleen, and cells lysates was determined using the BCA protein Assay Kit (ThermoFisher). Laemmli buffer to 1X final concentration (45 mM Tris-HCl-pH 6.8, 1.5% SDS, 3.5% β-mercaptoethanol, 3.5% Glycerol, 0.01% Bromophenol Blue) was added to equivalent protein amounts of cell lysates (5 μg for PBMCs lysates), tissue lysates (20 μg for muscle, spleen, liver, or tumor lysates) or lysate volumes (5 or 25 μl for IHLs lysates or muscle supernatants, respectively). To detect fully reduced and disulphide-HMGB1 isoforms, Western blot assays were performed in non-reducing conditions by diluting samples in Laemmli buffer without reducing agent (β-mercaptoethanol or DTT). Protein samples were separated on 14% SDS-PAGE (in reducing or non-reducing conditions) and transferred onto nitrocellulose membranes, which were blocked with 5% milk in Tris-buffered saline, pH 7.0, containing 0.1% Tween 20 (TBS-T). Membranes were probed with monoclonal rabbit anti-HMGB1 (1:10,000, EPR3507 Abcam) or rabbit anti-CD45 antibodies (1:500, ab10558 Abcam) in TBS-T plus 5% milk overnight at 4°C, washed several times with TBS-T, and incubated for 1 h with anti–rabbit peroxidase-conjugated antibody. For loading control, membranes were incubated with Ponceau Red (P7170 Sigma Aldrich) for a couple of minutes and then washed several times with TBS-T, or with monoclonal anti-GAPDH antibody (1:10,000, G9545 Sigma-Aldrich) in TBS-T plus 5% milk overnight at 4°C, washed several times with TBS-T, and incubated for 1 h with anti–rabbit Cy5-conjugated antibody. Western blots assays were visualized using a chemiluminescence kit or a Typhoon instrument according to the manufacturer's instructions (GE Healthcare).

### ELISA and Blood Analysis

Blood samples were collected, and serum was obtained by centrifugation for 10 min at 3,500 rpm at 4°C. The levels of HMGB1 protein were measured by ELISA (Tecan) according to manufacturer's instructions.

sALT levels were quantified in serum after APAP-induced intoxication with an International Federation of Clinical Chemistry and Laboratory Medicine-optimized kinetic UV method in an ILab Aries chemical analyzer (Instrumentation Laboratory).

### Statistical Analysis

Every experiment was replicated at least twice and was performed at least in biological triplicates. Sex-matched animals were assigned randomly to experimental groups and no animals were excluded from the study. According to the 3R rules, a power calculation analysis was previously performed. The evaluator was blinded to the identity of the specific sample as far as the nature of the experiment allowed it. Bars represent the mean ± SEM. Statistical significance was assessed by using the tests indicated in the figure legends (Prism 8; GraphPad Software). *P* < 0.05 were considered statistically significant.

## Results

### Redox Modulation of HMGB1 in Skeletal Muscle Upon Acute Injury

**Figure 1:**
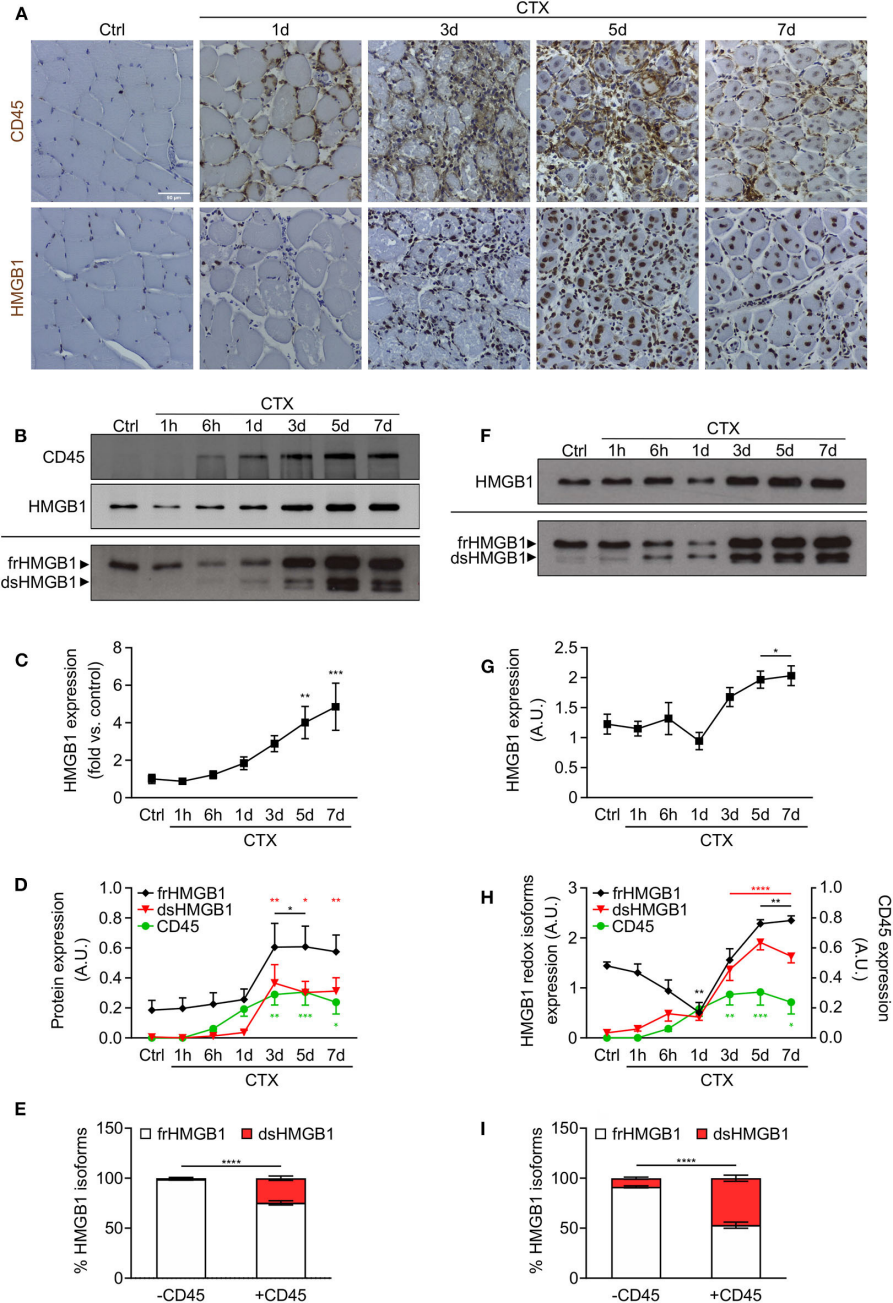
HMGB1 redox isoforms expression and leukocyte infiltration during acute muscle injury. **(A)** Representative images of immunohistochemical staining for CD45 (upper panel) and HMGB1 (lower panel) on tibialis anterior (TA) muscle sections at indicated time points after cardiotoxin (CTX) injection. Scale bars, 50 μm. Ctrl, uninjured control muscles. **(B–E)** Western blot probed with anti-CD45 (upper panel) and anti-HMGB1 (middle panel) antibodies in reducing conditions or with anti-HMGB1 antibody in non-reducing conditions (lower panel) on muscle lysates at indicated time points after CTX injection. The upper and lower bands in non-reducing conditions correspond to the fully reduced-HMGB1 (frHMGB1) and the disulphide-HMGB1 (dsHMGB1) isoforms, respectively. **(C)** Quantification of total HMGB1 protein expression levels, relative to control (Ctrl) and normalized on Ponceau staining, at indicated time points after CTX injection. A.U. = arbitrary unit (*n* ≥ 10 muscles, 3 mice/time point). **(D)** Quantification of CD45 and HMGB1 redox isoforms expression (frHMGB1 and dsHMGB1), normalized on Ponceau staining, at indicated time points. **(E)** Distribution of HMGB1 redox isoforms expression in muscle lysates in absence (controls and at 1 h post-injury) or presence of CD45-positive cells (from 6 h to day 7 post-injury). **(F–I)** Western blot probed with anti-HMGB1 antibody in reducing (upper panel) and non-reducing conditions (lower panel) on supernatant of muscles isolated at indicated time points after CTX injection **(F)**. Total HMGB1 protein expression at indicated time points after CTX injection **(G)**. A.U. = arbitrary unit (*n* = 6 muscle supernatants, 3 mice/time point). **(H)** Quantification of HMGB1 redox isoforms expression (frHMGB1 and dsHMGB1) in muscle supernatants, from Western blot assays in non-reducing conditions, at indicated time points after CTX injection and compared with CD45 expression as in **(D)**. **(I)** Distribution of HMGB1 redox isoforms expression in supernatants of muscle in absence (controls and at 1 h post-injury) or presence of CD45-positive cells (from 6 h to day 7 post-injury). Data represent the means ± SEM and statistical significance was calculated by One-way **(C,D,G,H)** and Two-way ANOVA **(E,I)**. *P < 0.05; **P < 0.01; ***P < 0.001; ****P < 0.0001.

Several reports highlighted a role of HMGB1 in skeletal muscle regeneration (17–19) and we previously demonstrated that HMGB1 redox isoforms orchestrate regeneration in muscle and liver after acute injury (13). HMGB1 is highly expressed in the nuclei of regenerating myofibers (Figure 1A) and both fr- and dsHMGB1 isoforms are abundant in the medium bathing injured muscles (7). We speculated that dsHMGB1 might derive from leukocytes infiltrating the injured muscle. To address this issue, we concomitantly analyzed the presence of leukocytes (CD45-positive cells) and the expression of HMGB1 redox isoforms in injured muscles at different time points after cardiotoxin (CTX)-induced acute injury. As evidenced by CD45 immunostaining on muscle sections and lysates, leukocytes were nearly undetectable in healthy muscle, and their infiltration started at 6 h post-injury and persisted until day 7 post-injury (Figures 1A,B). While the expression of total HMGB1 was increased from days 3–7 post-injury, both CD45-positive cells and dsHMGB1 appeared between 6 h and day 1 post-injury and persisted until day 7 post-injury (Figures 1B–D). The proportion of frHMGB1 on the total amount of HMGB1 decreased in muscle lysate from 6 h to day 7 post-injury and conversely, the proportion of dsHMGB1 was increased at these time points (Supplementary Figure 1A). Specifically, frHMGB1 was the isoform predominantly expressed at time points characterized by the absence of CD45-positive cells (control and 1 h post-injury), while dsHMGB1 represented about 30% of the total HMGB1 protein in muscle lysate in the presence of CD45-positive cells (from 6 h to day 7 post-injury) (Figure 1E).

HMGB1 is a marker of tissue damage as it is released by dead or stressed cells (6). In addition, leukocytes have been identified as professional cells for HMGB1 release upon injury and infection (9, 20). Hence, we analyzed the expression of released HMGB1 in serum and supernatant of injured muscles. Circulating HMGB1 was increased at early timepoints, from 1 to 6 h post-injury (Supplementary Figure 1B). This first peak of HMGB1 in the serum is most probably due to the release of the protein by necrotic cells from the injured muscle. To a lesser extent, we observed a second peak of HMGB1 from days 3–7 post-injury, which might be attributed to HMGB1 release by infiltrating leukocytes. The amount of HMGB1 was increased in supernatant of muscles excised from days 3–7 post-injury (Figures 1F,G). Notably, the expression of dsHMGB1 in the supernatant was much higher compared to those in muscle lysate and perfectly overlaps the expression of CD45 over time (Figures 1F–H, Supplementary Figure 1C). In the presence of CD45-positive cells, the percentage of dsHMGB1 on total HMGB1 protein level in the supernatant raised about 50% (Figure 1I).

Overall, our data demonstrate that the redox state of HMGB1 is highly modulated in skeletal muscle following acute injury, with a very low amount of dsHMGB1 in normal condition that strongly increases upon damage and is tightly associated with the presence of infiltrating leukocytes.

### Infiltrating and Circulating Leukocytes Represent Major Sources of dsHMGB1

**Figure 2:**
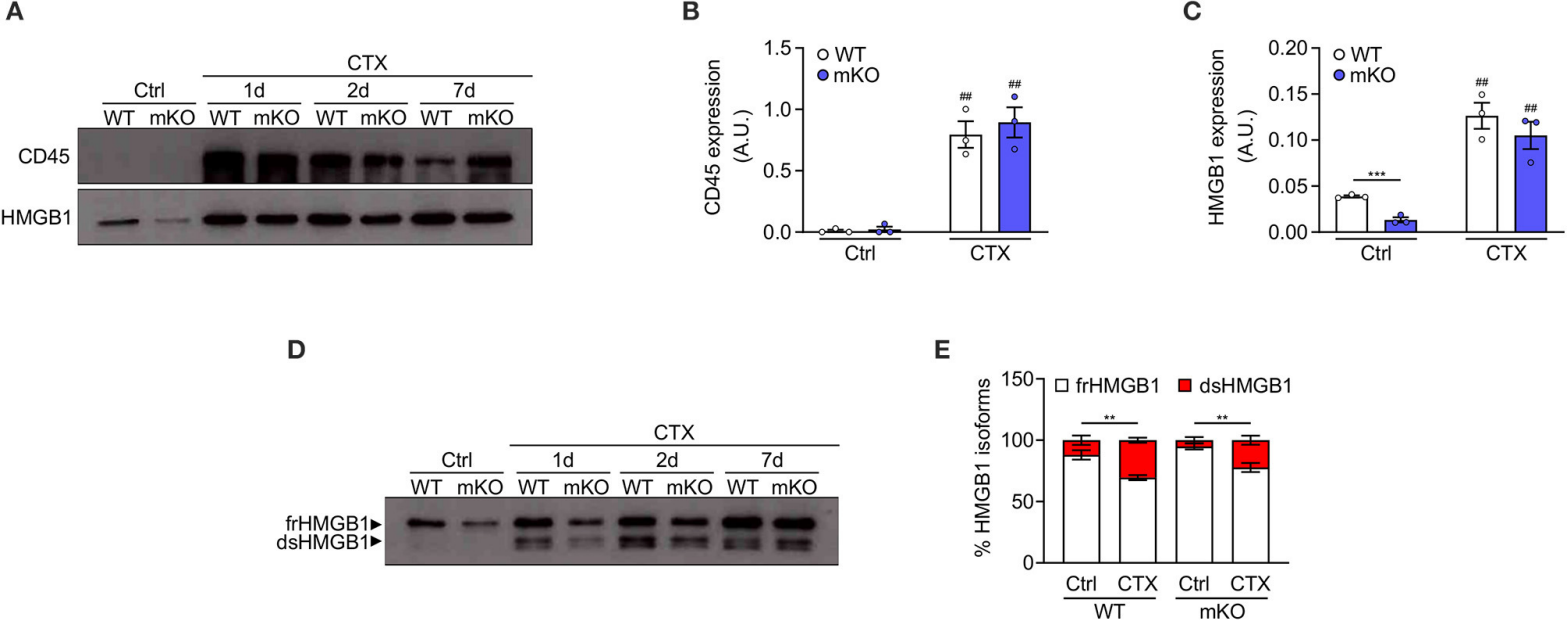
Disulphide-HMGB1 derives from non-myogenic cells in injured muscle. **(A)** Western blot probed with anti-CD45 (upper panel) and anti-HMGB1 (lower panel) antibodies in reducing conditions on tibialis anterior (TA) muscle lysates from WT or HMGB1 mKO mice at indicated time points after cardiotoxin (CTX) injection. Ctrl, control uninjured muscles. **(B,C)** Quantification of CD45 **(B)** and HMGB1 **(C)** protein expression, normalized on Ponceau staining, before (Ctrl) and after CTX injection (CTX at 1, 2, and 7 d) in TA and triceps muscle lysates (*n* ≥ 4 muscles/time point, *n* = 3 mice/genotype). A.U. = arbitrary unit. **(D,E)** Western blot probed with anti-HMGB1 antibody in non-reducing conditions **(D)** on TA muscle lysates from WT or HMGB1 mKO mice at indicated time points after CTX injection. The upper band corresponds to the fully reduced-HMGB1 (frHMGB1) and the lower band to the disulphide-HMGB1 (dsHMGB1). **(E)** Percentage of HMGB1 redox isoforms expression from WT or HMGB1 mKO mice before (Ctrl) and after CTX injection (CTX at 1, 2, 5, and 7 d) in TA and triceps muscle lysates (*n* ≥ 3 muscles/time point; *n* ≥ 4 mice/genotype). Data represent the means ± SEM and statistical significance was calculated by Student *T*-test **(B,C)** and Two-way ANOVA **(E)**. **P < 0.01; ***P < 0.001; ^*##*^*P* < 0.01 (Ctrl vs. CTX).

To demonstrate that dsHMGB1 derives from infiltrating leukocytes and not from resident muscle cells, we took advantage of muscle cells-specific HMGB1 knockout mice (hereafter mKO mice). These mice were generated by crossing C57BL/6 HMGB1 fl/fl mice with MyoD-Cre mice. In the latter, the Cre enzyme is under the control of the promoter of MyoD, a transcription factor expressed during myogenesis, which enables the deletion of lox-flanked sequences in all myogenic cells (muscle stem cells, myoblasts, myofibers). In this model, HMGB1 is deleted in myogenic cells but it is still expressed in skeletal muscle by non-muscle cell types, such as endothelial and nervous cells. As expected, the level of total HMGB1 was strongly decreased in uninjured muscle lysates from mKO mice compared to controls, and CD45-positive cells were nearly absent (Figures 2A–C). The level of HMGB1 and the number of CD45-positive cells strongly increased in both wildtype and mKO mice from day 1 to day 7 post-injury (Figures 2A–C), indicating that the absence of HMGB1 in myogenic cells does not dramatically affect leukocyte recruitment in injured muscle and that HMGB1 in injured muscle mainly derives from non-muscle cells. Similarly, we observed no difference in the distribution of HMGB1 redox isoforms between wildtype and HMGB1 mKO mice with detection of CD45 and dsHMGB1 only in injured muscles (Figures 2D,E). These data demonstrate that dsHMGB1 does not derive from myogenic cells in the injured muscle and strongly suggest that it originates from non-muscle cells such as infiltrating leukocytes.

**Figure 3:**
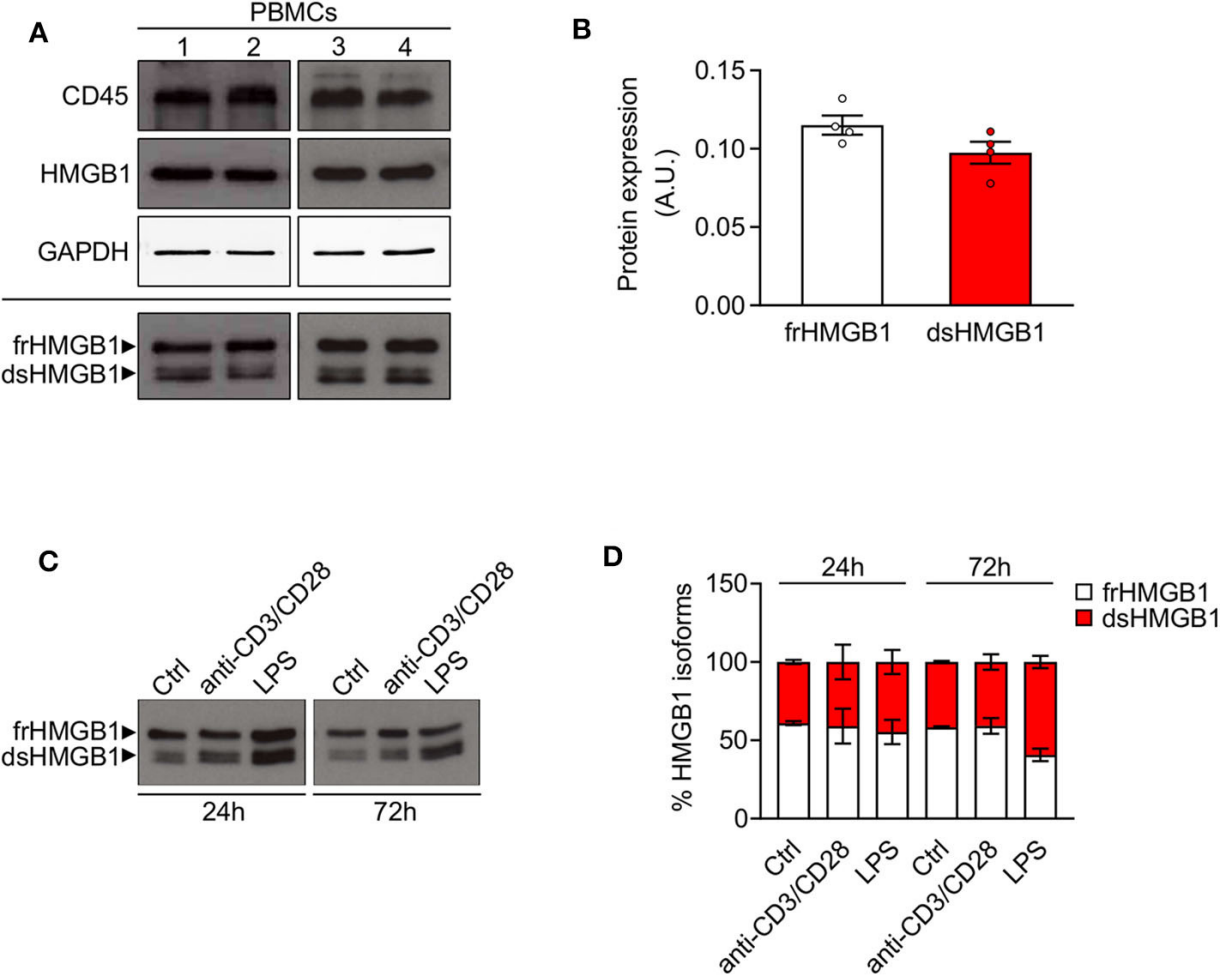
High expression of disulphide-HMGB1 in human leukocytes. **(A)** Western blot probed with anti-CD45, anti-HMGB1, and anti-GAPDH antibodies in reducing conditions (upper panels) or probed with anti-HMGB1 antibody in non-reducing conditions (lower panel) on peripheral blood mononuclear cells (PBMCs) isolated from four healthy human donors. The upper band corresponds to the fully reduced-HMGB1 (frHMGB1) and the lower band to the disulphide-HMGB1 (dsHMGB1) in the lower panel. **(B)** Quantification of HMGB1 redox isoforms expression normalized on Ponceau staining. A.U. = arbitrary unit (*n* = 4 healthy donors). **(C,D)** Western blot probed with anti-HMGB1 antibody in non-reducing conditions on PBMCs stimulated with anti-CD3/anti-CD28 antibodies or lipopolysaccharide (LPS) for 24 or 72 h **(C)**. Percentage of HMGB1 redox isoforms expression **(D)**. Ctrl, control unstimulated cells (*n* = 2 healthy donors). Data represent the means ± SEM and statistical significance was calculated by Two-way ANOVA **(D)**.

Although the release of HMGB1 by leukocytes has been widely studied, very little is known on the redox modulation of the protein in these cells. Hence, we analyzed the expression and redox state of HMGB1 in peripheral blood mononuclear cells (PBMCs) from human healthy donors. We observed a high expression of HMGB1 in lysate of freshly isolated PBMCs with around the 50% of the total corresponds to dsHMGB1 (Figures 3A,B), demonstrating that these cells represent a reservoir of dsHMGB1. To determine whether the expression and redox state of HMGB1 might be modulated during cell activation, we treated PBMCs with anti-CD3/anti-CD28 or lipopolysaccharide (LPS) to stimulate T cells, dendritic cells, and monocytes/macrophages. We observed a high amount of dsHMGB1 in all conditions with no major difference upon the various stimuli, although LPS appeared to slightly increase the level of dsHMGB1 at 72 h (Figures 3C,D).

Overall, our findings demonstrate that both circulating and infiltrating leukocytes contain a high amount of dsHMGB1 that could be released into the injured muscle.

### Leukocytes Operate as Transporters of dsHMGB1 in the Tumor Microenvironment

To further investigate the ability of leukocytes to transport dsHMGB1, we extended our results to cancer and to cachexia, a severe muscle wasting syndrome associated to tumor progression (21). Cancer-related inflammation has emerged as a hallmark of cancer and evidences from animal models indicate a compelling link between cachexia and inflammation (22, 23). Beside leukocyte invasion in the tumor microenvironment, cancer cachexia is associated to systemic inflammation, but no leukocyte infiltration in cachectic muscle (22, 24). To study the expression of HMGB1 redox isoforms in tumors and cachectic muscles, we employed two well-established mouse models of cancer cachexia: C57BL/6 and BalB/C mice injected subcutaneously with Lewis Lung Carcinoma (LLC) cells and colon adenocarcinoma C26 cells, respectively. In these models, mice undergo body weight loss and muscle wasting, mainly through increased levels of circulating Tumor Necrosis Factor-α and Interleukin-6, respectively (25, 26). We observed a loss of body weight and muscle mass in these two models, but the level of circulating HMGB1 was decreased in C26-bearing mice while it was increased in LLC-bearing mice (Supplementary Figures 2A–C), further underlining that tumor growth and cachexia progression are regulated by different mechanisms in these two models.

**Figure 4:**
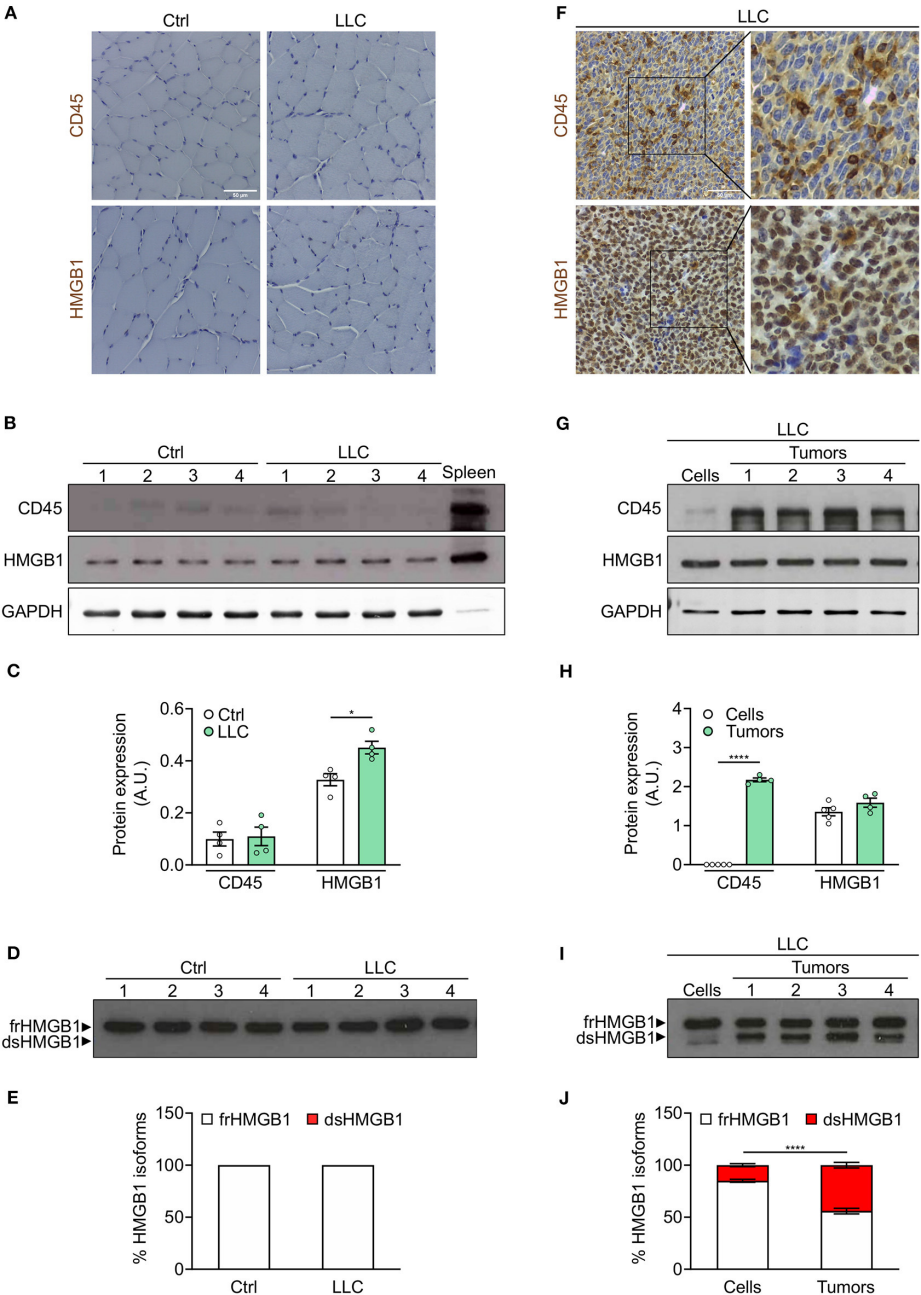
Leukocytes operate as transporter of dsHMGB1 in tumor microenvironment. **(A)** Representative images of immunohistochemical staining for CD45 (upper panel) and HMGB1 (lower panel) on tibialis anterior (TA) muscle sections from control (Ctrl) vs. Lewis lung carcinoma (LLC)-bearing mice. Scale bars, 50 μm. **(B,C)** Western blot probed with anti-CD45, anti-HMGB1, and anti-GAPDH antibodies in reducing conditions **(B)**, and quantification of total CD45 and HMGB1 protein levels normalized on GAPDH **(C)** (*n* = 4 mice). In **(B)**, spleen lysate (5 μg) was added as positive control for CD45 expression. **(D)** Western blot probed with anti-HMGB1 antibody in non-reducing conditions on tibialis anterior (TA) lysates from control or LLC-bearing mice. The upper and lower bands in non-reducing conditions correspond to the fully reduced-HMGB1 (frHMGB1) and the disulphide-HMGB1 (dsHMGB1) isoforms, respectively. **(E)** Percentage of HMGB1 redox isoforms expression. A.U. = arbitrary unit (*n* = 4 mice/group). **(F)** Immunohistochemical staining for CD45 (upper panel) and HMGB1 (lower panel) on tumoral sections from LLC-bearing mice. Scale bars, 50 μm. **(G–J)** Western blot probed with anti-CD45, anti-HMGB1, and anti-GAPDH antibodies in reducing conditions on LLC cells and tumoral masses isolated from mice injected with LLC cells **(G)**, and quantification of total CD45 and HMGB1 protein levels normalized on GAPDH **(H)**. **(I)** Western blot probed with anti-HMGB1 antibody in non-reducing conditions on LLC cells and tumoral masses isolated from mice injected with LLC cells. **(J)** Percentage of HMGB1 redox isoforms expression in LLC cultured cells and tumoral masses from LLC-injected mice **(J)**. A.U. = arbitrary unit (*n* = 5 cell replicates and *n* = 4 mice for tumoral masses). Data represent the means ± SEM and statistical significance was calculated by Student *T*-test **(C,H)** and Two-way ANOVA **(E,J)**. *P < 0.05; ****P < 0.0001.

As expected, CD45-positive cells were nearly absent in cachectic muscles (Figures 4A–C, Supplementary Figures 2D,E). We observed a slight increase of total HMGB1 level in cachectic muscles from both mouse models (Figures 4B,C, Supplementary Figures 2D,E). Interestingly, frHMGB1 was the predominant isoform with no increase of dsHMGB1 in cachectic muscles compared to controls (Figures 4D,E, Supplementary Figures 2F,G). These data demonstrate that leukocytes are not recruited and that the redox state of HMGB1 is not shifted toward dsHMGB1 in cachectic muscles.

We next analyzed the expression of CD45 and HMGB1 in tumors isolated from cachectic mice. Both CD45 and HMGB1 were highly expressed in LLC- and C26-derived tumors (Figures 4F–H, Supplementary Figures 2H,I). While the expression of total HMGB1 was comparable in isolated tumors and cultured tumor cell lines, CD45 and dsHMGB1 were highly expressed only in isolated tumors (Figures 4G–J, Supplementary Figures 2H–K).

Overall, these results demonstrate that the redox state of HMGB1 is modulated locally during cancer cachexia progression and indicate that leukocytes act as transporters of dsHMGB1 isoform in the tumor microenvironment.

### Redox Modulation of HMGB1 in Spleen and Liver

**Figure 5:**
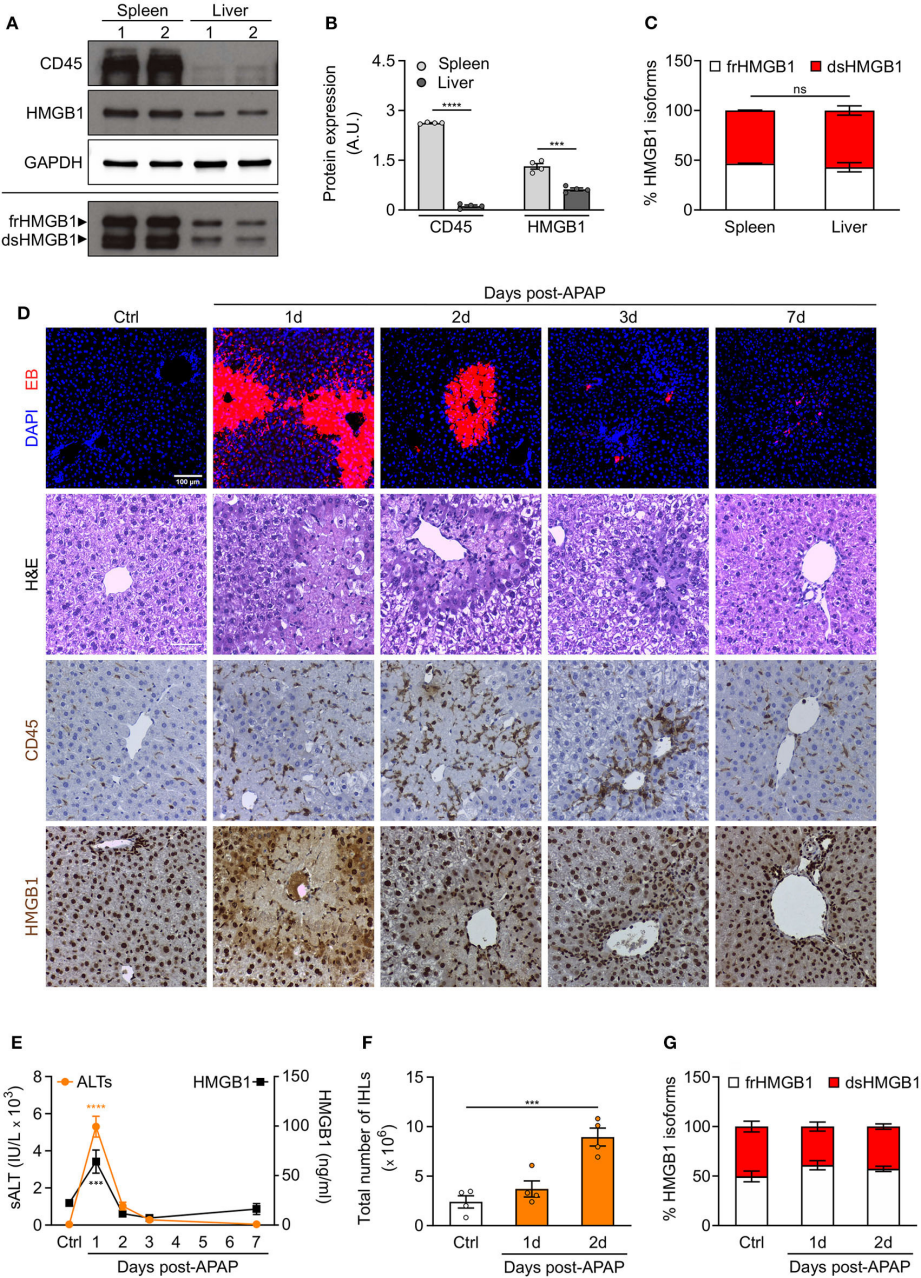
Redox modulation of HMGB1 in spleen and in drug-intoxicated liver. **(A)** Western blot probed with anti-CD45, anti-HMGB1, and anti-GAPDH antibodies in reducing conditions (upper panels) or probed with anti-HMGB1 antibody in non-reducing conditions (lower panel) on lysates of spleen and liver isolated from control WT mice. In the lower panel, the upper band corresponds to the fully reduced-HMGB1 (frHMGB1) and the lower band to the disulphide-HMGB1 (dsHMGB1). **(B,C)** Quantification of total CD45 and HMGB1 protein levels normalized on GAPDH **(B)**, and HMGB1 redox isoforms percentage **(C)** in spleen and liver lysates. A.U. = arbitrary unit (*n* = 4 mice/group). **(D–G)** Drug-induced liver injury (DILI) was induced by i.p. injection of acetaminophen (APAP), 300 mg/kg (body weight). Serum collection and necroscopy were performed at the indicated time points. **(D)** Representative images of DAPI and Evans Blue (EB) staining, Haematoxylin & Eosin (H&E) staining, and CD45 and HMGB1 immunostaining in liver sections from control mice (Ctrl) and at days 1, 2, 3, and 7 after DILI. Scale bars, 50 μm. **(E)** Alanine aminotransferase (sALT) and HMGB1 levels in serum before and after APAP injection in mice (*n* ≥ 5 mice/group). **(F)** Quantification of total number of intrahepatic leukocytes (IHLs) in control mice and at days 1 and 2 post-APAP injection (*n* = 4 mice/group). **(G)** Quantification of HMGB1 redox isoforms percentage, from Western blot assays performed in non-reducing conditions with anti-HMGB1 antibody, in IHLs isolated from control mice and at days 1 and 2 post-APAP injection (*n* = 4 mice/group). Data represent the means ± SEM and statistical significance was calculated by Student *T*-test **(B)**, One-way **(E,F)** and Two-way ANOVA **(C,G)**. ***P < 0.001; ****P < 0.0001; ns, not significant.

To determine whether resident leukocytes, as opposed to infiltrating/circulating leukocytes, also produce dsHMGB1, we analyzed the expression of HMGB1 redox isoforms in spleen and liver, two organs characterized by a high number of resident leukocytes. We observed a high expression of both CD45 and dsHMGB1 in spleen whereas comparable percentage of dsHMGB1 expression was associated to much lower CD45 expression in liver (Figures 5A–C), suggesting additional cell population(s) expressing dsHMGB1 in liver. Beside differences in leukocytes number, these findings indicate that these two organs represent important sources of dsHMGB1.

Previous studies have demonstrated that endogenous HMGB1 is a mediator of drug-induced hepatoxicity by promoting inflammation via its interaction with TLR4/MD-2 (14). We decided to analyse the recruitment of leukocytes and the expression of HMGB1 redox isoforms in liver upon acetaminophen (APAP) intoxication. We hypothesized that no difference in dsHMGB1 percentage would be observed in liver upon acute injury as we already observed a comparable percentage of dsHMGB1 in circulating leukocytes and uninjured liver (around 50% of total HMGB1). As expected, we observed hepatocyte necrosis, CD45-positive cell recruitment and HMGB1 release in injured areas of the liver after APAP injection (Figure 5D). Notably, the temporal dynamics of circulating HMGB1 levels perfectly overlaps that of sALT, a marker of liver damage (Figure 5E), which peaks at day 1 post-APAP. As previously described (13), the peak of intrahepatic leukocytes (IHLs) occurs at day 2 post-APAP (Figures 5D,F). We performed Western blot analyses on IHLs isolated from liver, and we observed that both resident and infiltrating leukocytes upon APAP intoxication express high level of dsHMGB1 (Figure 5G, Supplementary Figure 3A). As expected, although the number of CD45 positive cells was higher in APAP-treated mice, the percentage of dsHMGB1 in total liver lysate was similar in control and intoxicated mice (Supplementary Figures 3B–D), indicating that cell population(s) distinct from leukocytes might also produce dsHMGB1 in liver.

Overall, these findings demonstrate that spleen and liver express high level of dsHMGB1 in physiological conditions, and that both resident/infiltrating leukocytes and additional uncharacterized cell population(s) are sources of dsHMGB1 in normal and intoxicated livers.

## Discussion

Although it is well-established that HMGB1 is a critical mediator of inflammation and is involved in numerous inflammatory disorders, clinical trials to specifically target the protein are still to come. A deeper understanding of both intracellular and extracellular functions of HMGB1 is essential to develop efficient therapeutic interventions targeting this alarmin. In this context, the discovery of HMGB1 redox modulation represented a breakthrough in the field, and our findings now reveal a highly dynamic regulation of HMGB1 oxidation *in vivo*, both upon tissue injury and in the tumor microenvironment, which is tightly associated to inflammatory processes. In addition, we identified the leukocyte cell population as a reservoir and transporter of dsHMGB1.

A growing body of evidence indicates that frHMGB1 orchestrates cell recruitment and tissue regeneration while dsHMGB1 contributes to inflammation by activating immune cells (11–13). However, most studies were performed using recombinant HMGB1 redox isoforms. Here, we analyzed the dynamics of expression of endogenous HMGB1 isoforms, demonstrating that the redox state of HMGB1 is highly modulated *in vivo* in different tissues, both in physiological and pathological conditions. Indeed, our results indicate that the redox modulation of HMGB1 is tissue-specific, with a high expression of dsHMGB1 in normal conditions in spleen or liver while it is almost absent in skeletal muscle. Similarly, dsHMGB1 is highly expressed in the tumor microenvironment while it is absent in cachectic muscles from the same tumor-bearing mice. It is well-established that cancer cachexia is characterized by systemic inflammation and leukocytes infiltration in the tumor, but not in the cachectic muscles (24). Accordingly, we observed a high expression of both CD45 and dsHMGB1 in tumors, but not in cachectic muscles. Hence, these data clearly establish that the redox state of HMGB1 is locally controlled and demonstrate that the presence of dsHMGB1 is tightly associated with leukocytes infiltration.

Besides being spatially restricted, HMGB1 oxidation is regulated in time. In skeletal muscle, dsHMGB1 appears a couple of hours after an acute injury. In liver, dsHMGB1 is highly expressed both at basal level and upon drug intoxication, indicating that cell populations other than leukocytes might contribute to the production of dsHMGB1 in liver. Overall, our findings point out to an accurate and dynamic regulation of HMGB1 redox state in physiological and pathological conditions, most probably to finetune the inflammatory and regenerative processes.

Extracellular HMGB1 has been identified as a drug-target protein in multiple diseases, in particular in inflammation-associated disorders, and as a target of aspirin (27), the most widely used drug worldwide, and of the salicylate diflunisal (28), demonstrating the importance of HMGB1 in clinic. A high level of serum HMGB1 appears to be a sensitive biomarker in diverse disorders, such as mesothelioma, but the different HMGB1 isoforms represent novel biomarker candidates that provide additional mechanistic information (29). Indeed, total HMGB1 is indicative of both cell death and immune cell activation while the characterization of the oxidation state can provide pivotal information on the type of injury and on inflammation degree. So far, it is not possible to detect the different isoforms by ELISA assay due to the difficulty to generate antibodies specific for each isoform. Mass spectrometry analyses have been widely employed to analyse the posttranslational modifications of HMGB1 such as acetylation and oxidation. However, this methodology is costly and time-consuming, considerably limiting its potential application in the clinic. Iwahara et al., proposed an NMR-based approach to study the kinetics of HMGB1 oxidation in extracellular fluids (30). Although this technique has multiple advantages, it can be applied only to extracellular fluids. In our study, we showed that it is possible to perform Western blot assays to analyse the expression of HMGB1 redox isoforms both at cell and tissue levels. Other studies reported the detection of HMGB1 redox isoforms in serum and plasma by Western blot assay (31, 32), showing that this method can also be applied to extracellular fluids. Western blot has a wide range of applications in the clinic, such as the application of medical diagnosis for infectious diseases including hepatitis C (HCV), HIV, Lyme disease, and syphilis, as well as autoimmune disorders such as paraneoplastic disease and myositis (33). In conclusion, the analysis of HMGB1 redox isoforms expression by Western blot assay might be useful not only for research but also for clinical applications.

An important issue to address is to determine in which conditions HMGB1 gets oxidized and if its oxidation occurs outside and/or inside the cells. HMGB1 is secreted through a non-classical vesicle-mediated secretory pathway, bypassing the endoplasmic reticulum (ER) (20). The redox potential of the ER is continually preserved as an oxidizing environment to facilitate the oxidative process of disulphide bond formation during protein folding. Hence, the avoidance of the ER limits HMGB1 oxidation. Conversely, a recent study indicates that HMGB1 oxidation can occur in the nucleus of mouse bone marrow-derived macrophages, mouse embryonic fibroblasts and HEK293T cells (34). The authors demonstrate that disulphide bond formation is required for HMGB1 nucleocytoplasmic translocation and secretion, and is mediated by peroxiredoxins (Prxs), a ubiquitous family of antioxidant enzymes highly expressed in cells. We observed high levels of dsHMGB1 in lysates of leukocytes from mice and healthy donors, demonstrating that HMGB1 was already oxidized inside the cells. Indeed, it is well-known that leukocytes produce Reactive Oxygen Species (ROS) as part of the killing response against microbial invasion and as intra- and intercellular messengers. Conversely, a recent study showed that HMGB1 is maintained in a reduced state, owing to the activity of the thioredoxin antioxidant system, in monocytes from patients with active rheumatoid arthritis (35). Future investigation should characterize the molecular mechanisms driving HMGB1 oxidation in extracellular and intracellular spaces, in particular in leukocytes.

Inflammatory conditions are associated with the release of ROS in the microenvironment, in particular by leukocytes. Hence, HMGB1 is most probably oxidized also when it is present in the extracellular space, especially in inflammatory conditions. Accordingly, it has been reported that HMGB1 is rapidly oxidized in the extracellular space and that the half-life for frHMGB1 is 17 min *in vitro* in serum before it gets converted to dsHMGB1 (30). The authors showed large variations in the kinetics for HMGB1 oxidation and clearance in different extracellular fluids, clearly demonstrating that the balance between fr- and dsHMGB1 depends on the extracellular environment. Similarly, we observed higher level of dsHMGB1 in supernatants than in lysates of injured muscles, suggesting that HMGB1 was partially oxidized outside the cells. Most importantly, our results identify leukocytes as a source of dsHMGB1 in muscle, spleen, liver, and tumor. These findings are relevant because they demonstrate that leukocytes can also operate as vehicle of dsHMGB1 in the tissue. However, the relative contribution of non-muscle cell types (e.g., endothelial cells) resident in skeletal muscle to HMGB1 release and redox regulation is still unknown. Similarly, the high expression of dsHMGB1 in healthy liver suggests that it originates from a cell population different from leukocytes. Hence, further investigation is required to decipher the regulation of HMGB1 redox state at both tissue and cell levels.

Overall, our study underlines a close association of dsHMGB1 expression with an inflammatory state characterized by immune cells presence, and identifies leukocytes as reservoirs and transporters of dsHMGB1. These findings emphasize that HMGB1 oxidation is a timely and spatially regulated process in physiological and pathological conditions, most likely to finetune inflammatory and regenerative processes.

## Data Availability Statement

All datasets presented in this study are included in the article/Supplementary Material.

## Ethics Statement

The animal study was reviewed and approved by the San Raffaele Institutional Animal Care and Use Committee.

## Author Contributions

MF and GCh designed, carried out most of the experiments and analyzed data. LF and GS designed and carried out experiments of acetaminophen intoxication in mice, and analyzed data. GCa and ER contributed to design, carry out experiments of acute muscle injury in mice, and analyze data. AP performed ELISA for HMGB1. RP provided advice for design and technical details of experiments of cancer cachexia. MB discussed results and provided advice on experimental design. EV designed experiments, directed the project and wrote the manuscript with comments from all authors. All authors contributed to the article and approved the submitted version.

## Conflict of Interest

MB is founder and part owner of HMGBiotech, a company that provides goods and services related to HMGB proteins, and AP is partially supported by HMGBiotech. The remaining authors declare that the research was conducted in the absence of any commercial or financial relationships that could be construed as a potential conflict of interest. The reviewer UA declared a past collaboration with one of the authors MB to the handling Editor.

## Supplementary Material

Supplementary Figure 1 HMGB1 redox isoforms expression in muscle and HMGB1 level in serum upon acute muscle injury. **(A)** Percentage of HMGB1 redox isoforms expression, quantified from Western blot assays performed in non-reducing conditions with anti-HMGB1 antibody, on tibialis anterior (TA) and triceps muscle lysates at indicated time points after cardiotoxin (CTX) injection. A.U. = arbitrary unit (*n* = 11 muscles, 3 mice/time point). **(B)** Quantification of HMGB1 protein level (ng/ml) by ELISA in the serum of control (Ctrl) and CTX-treated mice at indicated time points (*n* ≥ 3 mice/time points). **(C)** Percentage of HMGB1 redox isoforms expression, quantified from Western blot assays performed in non-reducing conditions with anti-HMGB1 antibody, on muscle supernatants at indicated time points after CTX injection. A.U. = arbitrary unit (*n* = 6 muscle supernatants, 3 mice/time point). Data represent the means ± SEM and statistical significance was calculated by One-way ANOVA **(A–C)**. ^*^*P* < 0.05; ^**^*P* < 0.01; ^***^*P* < 0.001; ^****^*P* < 0.0001.

Supplementary Figure 2 Redox modulation of HMGB1 during cancer cachexia. **(A)** Body weight (g) of mice injected with LLC or C26 cells at day 0 or at the endpoint of the experiment. **(B)** Weight loss percentage of gastrocnemius (GAS), tibialis anterior (TA), and quadriceps (QUAD) muscles from LLC- or C26-bearing mice (*n* ≥ 4 mice/group). **(C)** Quantification of HMGB1 protein level (ng/ml) by ELISA in the serum of control (Ctrl) or tumor-bearing mice (LLC or C26) (*n* ≥ 4 mice/group). **(D,E)** Western blot probed with anti-CD45, anti-HMGB1, and anti-GAPDH antibodies in reducing conditions **(D)** on tibialis anterior (TA) muscle lysates from control (Ctrl) or C26-bearing mice. In **(D)**, spleen lysate (5 μg) was added as positive control for CD45 expression. **(E)** Quantification of total CD45 and HMGB1 protein levels normalized on GAPDH. A.U. = arbitrary unit (*n* ≥ 4 mice/group). **(F,G)** Western blot probed with anti-HMGB1 antibody in non-reducing conditions on TA muscles isolated from control or C26-bearing mice **(F)**. The upper and lower bands correspond to the fully-reduced HMGB1 (frHMGB1) and the disulphide-HMGB1 (dsHMGB1) isoforms, respectively. **(G)** Quantification of HMGB1 redox isoforms percentage. A.U. = arbitrary unit (*n* ≥ 4 mice/group). **(H,I)** Western blot probed with anti-CD45, anti-HMGB1, and anti-GAPDH antibodies in reducing conditions on C26 cultured cells (Cells) and on tumoral masses (Tumors) isolated from mice injected with C26 cells **(H)**. Quantification of total CD45 and HMGB1 protein expression normalized on GAPDH **(I)**. A.U. = arbitrary unit (*n* = 4 cell replicates and *n* = 5 mice for tumoral masses). **(J,K)** Western blot probed with anti-HMGB1 antibody in non-reducing conditions on cultured C26 cells (Cells) and on tumoral masses (Tumors) isolated from C26-bearing mice **(J)**. Quantification of HMGB1 redox isoforms percentage in cultured C26 cells (Cells) and in tumoral masses (Tumors) isolated from C26-injected mice (**K**; *n* = 4 cell replicates and *n* = 5 mice for tumoral masses). Data represent the means ± SEM and statistical significance was calculated by Student *T*-test **(A,C,E,I)** and Two-way ANOVA **(G,K)**. ^*^*P* < 0.05; ^**^*P* < 0.01; ^***^*P* < 0.001; ^****^*P* < 0.0001; ns, not significant.

Supplementary Figure 3 CD45 and HMGB1 redox isoforms expression in liver after drug intoxication. Drug-induced liver injury (DILI) was induced by i.p. injection of acetaminophen (APAP), 300 mg/kg (body weight). Liver and intrahepatic leukocytes (IHLs) isolations were performed at indicated time points after APAP treatment. **(A)** Western blot probed with anti-CD45, anti-HMGB1, and anti-GAPDH antibodies in reducing conditions (upper panels) or probed with anti-HMGB1 antibody in non-reducing conditions (lower panel) on IHLs isolated from control (Ctrl) and APAP-treated mice at indicated time points. In the lower panel, the upper band corresponds to the fully-reduced HMGB1 (frHMGB1) and the lower band to the disulphide-HMGB1 (dsHMGB1). **(B)** Western blot probed with anti-CD45, anti-HMGB1, and anti-GAPDH antibodies in reducing conditions (upper panels) or probed with anti-HMGB1 antibody in non-reducing conditions (lower panel) on liver lysates of control (Ctrl) and APAP-injected mice at indicated time points. **(C)** Quantification of total CD45 and HMGB1 protein expression in control (Ctrl) and APAP-treated mice at indicated time points. A.U. = arbitrary unit (*n* = 4 mice/group). **(D)** Quantification of HMGB1 redox isoforms percentage in liver lysates from control (Ctrl) and APAP-treated mice (*n* = 4 mice/group). Data represent the means ± SEM and statistical significance was calculated by One-way **(C)** and Two-way ANOVA **(D)**. ^**^*P* < 0.01; ^****^*P* < 0.0001.

## References

[1] Karin M Clevers H. Reparative inflammation takes charge of tissue regeneration. Nature. (2016) 529:307–15. 10.1038/nature17039 26791721 PMC5228603

[2] Oppenheim JJ Yang D. Alarmins: chemotactic activators of immune responses. Curr Opin Immunol. (2005) 17:359–65. 10.1016/j.coi.2005.06.002 15955682

[3] Vénéreau E Ceriotti C Bianchi ME. DAMPs from cell death to new life. Front. Immunol. (2015) 6:422. 10.3389/fimmu.2015.00422 26347745 PMC4539554

[4] Celona B Weiner A Di Felice F Mancuso FM Cesarini E Rossi RL. Substantial histone reduction modulates genomewide nucleosomal occupancy and global transcriptional output. PLoS Biol. (2011) 9:e1001086. 10.1371/journal.pbio.1001086 21738444 PMC3125158

[5] Agresti A Bianchi ME. HMGB proteins and gene expression. Curr Opin Genet Dev. (2003) 13:170–8. 10.1016/S0959-437X(03)00023-6 12672494

[6] Scaffidi P Misteli T Bianchi ME. Release of chromatin protein HMGB1 by necrotic cells triggers inflammation. Nature. (2002) 418:191–5. 10.1038/nature00858 12110890

[7] Venereau E Casalgrandi M Schiraldi M Antoine DJ Cattaneo A De Marchis F Mutually exclusive redox forms of HMGB1 promote cell recruitment or proinflammatory cytokine release. J Exp Med. (2012) 209:1519–28. 10.1084/jem.20120189 22869893 PMC3428943

[8] Venereau E Schiraldi M Uguccioni M Bianchi ME. HMGB1 and leukocyte migration during trauma and sterile inflammation. Mol. Immunol. (2013) 55:76–82. 10.1016/j.molimm.2012.10.037 23207101

[9] Wang H Bloom O Zhang M Vishnubhakat JM Ombrellino M Che J. HMG-1 as a late mediator of endotoxin lethality in mice. Science. (1999) 285:248–51. 10398600 10.1126/science.285.5425.248

[10] Di Candia L Gomez E Venereau E Chachi L Kaur D Bianchi ME. HMGB1 is upregulated in the airways in asthma and potentiates airway smooth muscle contraction via TLR4. J Allergy Clin Immunol. (2017) 140:584–87.e8. 10.1016/j.jaci.2016.11.049 28259445 PMC5540224

[11] Yang H Lundbäck P Ottosson L Erlandsson-Harris H Venereau E Bianchi ME. Redox modification of cysteine residues regulates the cytokine activity of high mobility group box-1 (HMGB1). Mol Med. (2012) 18:250–9. 10.2119/molmed.2011.00389 22105604 PMC3324950

[12] Schiraldi M Raucci A Muñoz LM Livoti E Celona B Venereau E. HMGB1 promotes recruitment of inflammatory cells to damaged tissues by forming a complex with CXCL12 and signaling via CXCR4. J Exp Med. (2012) 209:551–63. 10.1084/jem.20111739 22370717 PMC3302219

[13] Tirone M Tran NL Ceriotti C Gorzanelli A Canepari M Bottinelli R. High mobility group box 1 orchestrates tissue regeneration via CXCR4. J Exp Med. (2018) 215:303–18. 10.1084/jem.20160217 29203538 PMC5748844

[14] Yang H Wang H Ju Z Ragab AA Lundbäck P Long W. MD-2 is required for disulfide HMGB1-dependent TLR4 signaling. J Exp Med. (2015) 212:5–14. 10.1084/jem.20141318 25559892 PMC4291531

[15] Hoppe G Talcott KE Bhattacharya SK Crabb JW Sears JE. Molecular basis for the redox control of nuclear transport of the structural chromatin protein Hmgb1. Exp. Cell Res. (2006) 312:3526–38. 10.1016/j.yexcr.2006.07.020 16962095

[16] Sitia G Iannacone M Müller S Bianchi ME Guidotti LG. Treatment with HMGB1 inhibitors diminishes CTL-induced liver disease in HBV transgenic mice. J Leukoc Biol. (2007) 81:100–7. 10.1189/jlb.0306173 16935945

[17] Campana L Santarella F Esposito A Maugeri N Rigamonti E Monno A. Leukocyte HMGB1 is required for vessel remodeling in regenerating muscles. J Immunol. (2014) 192:5257–64. 10.4049/jimmunol.1300938 24752445

[18] De Mori R Straino S Di Carlo A Mangoni A Pompilio G Palumbo R. Multiple effects of high mobility group box protein 1 in skeletal muscle regeneration. Arterioscler Thromb Vasc Biol. (2007) 27:2377–83. 10.1161/ATVBAHA.107.153429 17872450

[19] Dormoy-Raclet V Cammas A Celona B Lian XJ van der Giessen K Zivojnovic M. HuR and miR-1192 regulate myogenesis by modulating the translation of HMGB1 mRNA. Nat Commun. (2013) 4:2388. 10.1038/ncomms3388 24005720 PMC4005793

[20] Gardella S Andrei C Ferrera D Lotti LV Torrisi MR Bianchi ME. The nuclear protein HMGB1 is secreted by monocytes via a non-classical, vesicle-mediated secretory pathway. EMBO Rep. (2002) 3:995–1001. 10.1093/embo-reports/kvf198 12231511 PMC1307617

[21] Baracos VE Martin L Korc M Guttridge DC Fearon KCH. (2018). Cancer-associated cachexia. Nat Rev Dis Primers. 4:17105. 10.1038/nrdp.2017.105 29345251

[22] Argiles JM Lopez-Soriano FJ Busquets S. Counteracting inflammation: a promising therapy in cachexia. Crit Rev Oncog. (2012) 17:253–62. 10.1615/CritRevOncog.v17.i3.30 22831156

[23] Argilés JM Busquets S Stemmler B López-Soriano FJ. Cancer cachexia: understanding the molecular basis. Nat Rev Cancer. (2014) 14:754–62. 10.1038/nrc3829 25291291

[24] Berardi E Aulino P Murfuni I Toschi A Padula F Scicchitano BM. Skeletal muscle is enriched in hematopoietic stem cells and not inflammatory cells in cachectic mice. Neurol Res. (2008) 30:160–9. 10.1179/174313208X281046 18397608

[25] Deboer MD. Animal models of anorexia and cachexia. Expert Opin Drug Discov. (2009) 4:1145–55. 10.1517/17460440903300842 20160874 PMC2771941

[26] Martinelli GB Olivari D Re Cecconi AD Talamini L Ottoboni L Lecker SH. Activation of the SDF1/CXCR4 pathway retards muscle atrophy during cancer cachexia. Oncogene. (2016) 35:6212–22. 10.1038/onc.2016.153 27212031

[27] Choi HW Tian M Song F Venereau E Preti A Park SW. Aspirin's active metabolite salicylic acid targets high mobility group box 1 to modulate inflammatory responses. Mol Med. (2015) 21:526–35. 10.2119/molmed.2015.00148 26101955 PMC4607614

[28] De Leo F Quilici G Tirone M De Marchis F Mannella V Zucchelli C. Diflunisal targets the HMGB1/CXCL12 heterocomplex and blocks immune cell recruitment. EMBO Rep. (2019) 20:e47788. 10.15252/embr.201947788 31418171 PMC6776901

[29] Venereau E De Leo F Mezzapelle R Careccia G Musco G Bianchi ME. HMGB1 as biomarker and drug target. Pharmacol. Res. (2016) 111:534–44. 10.1016/j.phrs.2016.06.031 27378565

[30] Zandarashvili L Sahu D Lee K Lee YS Singh P Rajarathnam K. Real-time kinetics of high-mobility group box 1 (HMGB1) oxidation in extracellular fluids studied by in situ protein NMR spectroscopy. J Biol Chem. (2013) 288:11621–7. 10.1074/jbc.M113.449942 23447529 PMC3636853

[31] Kim ID Lee H Kim SW Lee HK Choi J Han PL. Alarmin HMGB1 induces systemic and brain inflammatory exacerbation in post-stroke infection rat model. Cell Death Dis. (2018) 9:426. 10.1038/s41419-018-0438-8 29555931 PMC5859283

[32] Urbonaviciute V Meister S Fürnrohr BG Frey B Gückel E Schett G. Oxidation of the alarmin high-mobility group box 1 protein (HMGB1) during apoptosis. Autoimmunity. (2009) 42:305–7. 10.1080/08916930902831803 19811284

[33] Gorr TA Vogel J. Western blotting revisited: critical perusal of underappreciated technical issues. Proteomics Clin Appl. (2015) 9:396–405. 10.1002/prca.201400118 25597284

[34] Kwak MS Kim HS Lkhamsuren K Kim YH Han MG Shin JM. Peroxiredoxin-mediated disulfide bond formation is required for nucleocytoplasmic translocation and secretion of HMGB1 in response to inflammatory stimuli. Redox Biol. (2019) 24:101203. 10.1016/j.redox.2019.101203 31026770 PMC6482348

[35] Cecchinato V D'Agostino G Raeli L Nerviani A Schiraldi M Danelon G. Redox-mediated mechanisms fuel monocyte responses to CXCL12/HMGB1 in active rheumatoid arthritis. Front Immunol. (2018) 9:2118. 10.3389/fimmu.2018.02118 30283452 PMC6157448

