# Supplementary figures and images for: Oxidation of HMGB1 Is a Dynamically Regulated Process in Physiological and Pathological Conditions
Source: Front Immunol. 2020 Jun 24;11:1122. doi: 10.3389/fimmu.2020.01122 (PMC7326777; doi:10.3389/fimmu.2020.01122)

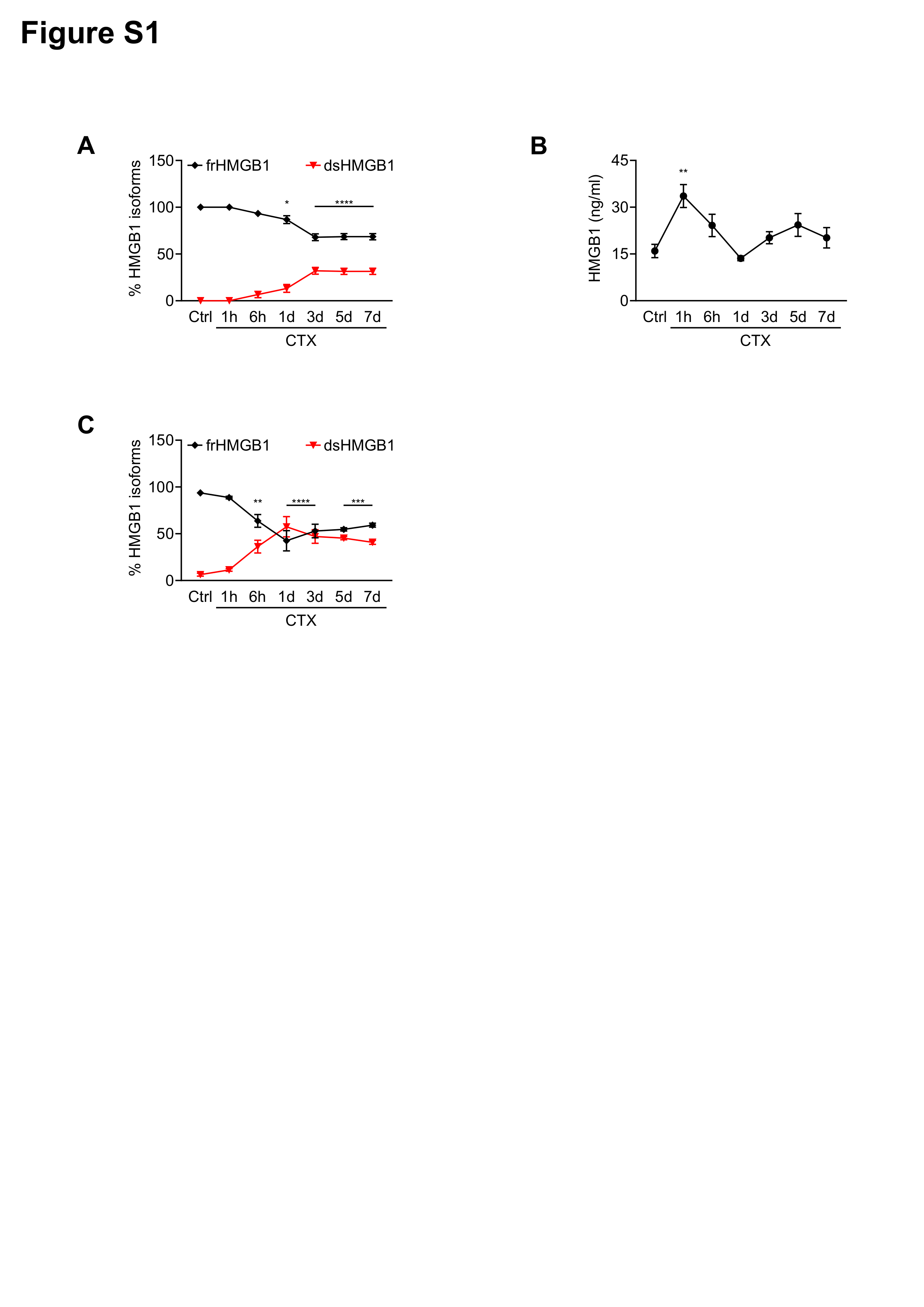

Supplement: Supplementary Figure 1 — HMGB1 redox isoforms expression in muscle and HMGB1 level in serum upon acute muscle injury. (A) Percentage of HMGB1 redox isoforms expression, quantified from Western blot assays performed in non-reducing conditions with anti-HMGB1 antibody, on tibialis anterior (TA) and triceps muscle lysates at indicated time points after cardiotoxin (CTX) injection. A.U. = arbitrary unit (n = 11 muscles, 3 mice/time point). (B) Quantification of HMGB1 protein level (ng/ml) by ELISA in the serum of control (Ctrl) and CTX-treated mice at indicated time points (n ≥ 3 mice/time points). (C) Percentage of HMGB1 redox isoforms expression, quantified from Western blot assays performed in non-reducing conditions with anti-HMGB1 antibody, on muscle supernatants at indicated time points after CTX injection. A.U. = arbitrary unit (n = 6 muscle supernatants, 3 mice/time point). Data represent the means ± SEM and statistical significance was calculated by One-way ANOVA (A–C). *P < 0.05; **P < 0.01; ***P < 0.001; ****P < 0.0001. [file Image_1.tiff]

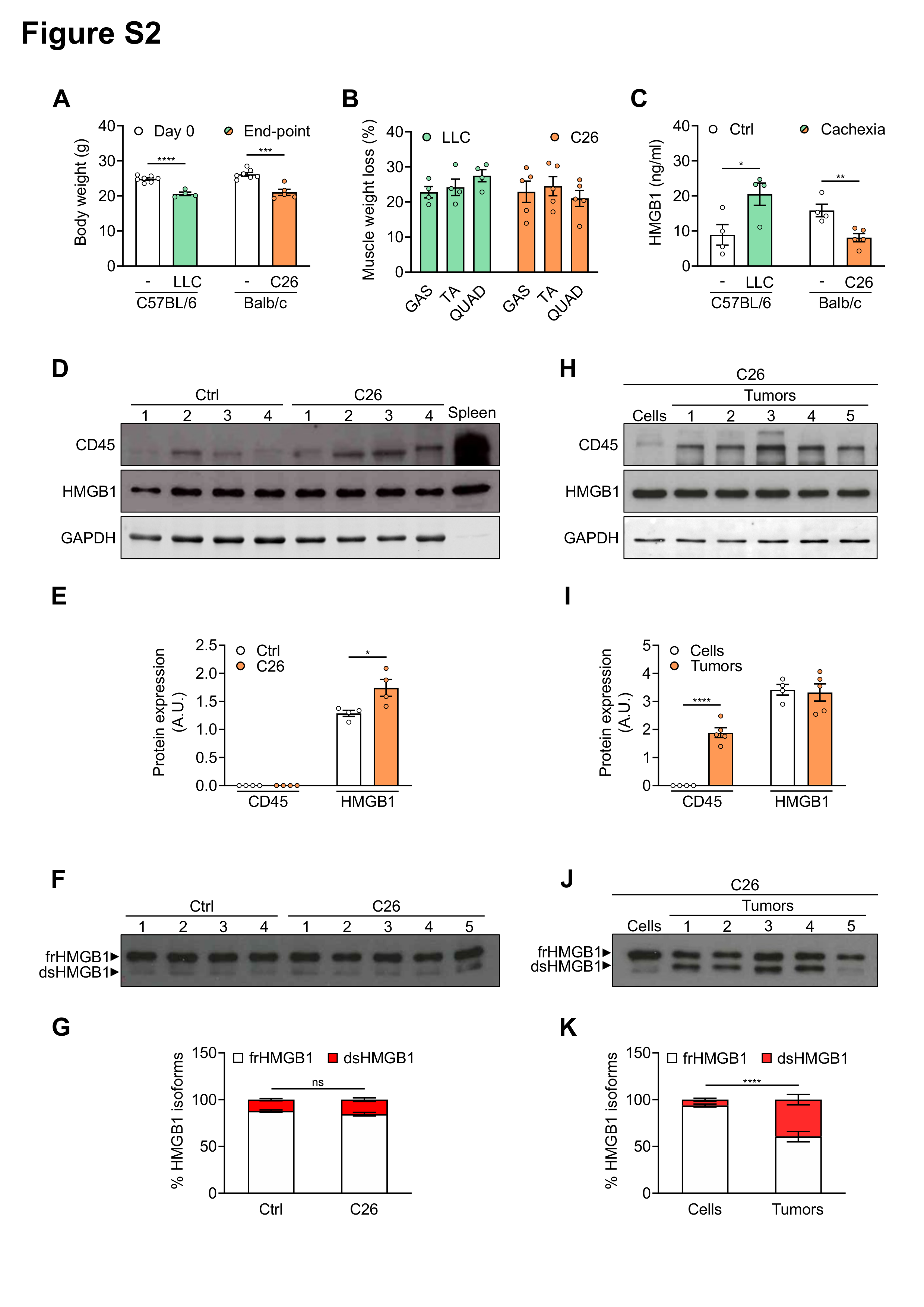

Supplement: Supplementary Figure 2 — Redox modulation of HMGB1 during cancer cachexia. (A) Body weight (g) of mice injected with LLC or C26 cells at day 0 or at the endpoint of the experiment. (B) Weight loss percentage of gastrocnemius (GAS), tibialis anterior (TA), and quadriceps (QUAD) muscles from LLC- or C26-bearing mice (n ≥ 4 mice/group). (C) Quantification of HMGB1 protein level (ng/ml) by ELISA in the serum of control (Ctrl) or tumor-bearing mice (LLC or C26) (n ≥ 4 mice/group). (D,E) Western blot probed with anti-CD45, anti-HMGB1, and anti-GAPDH antibodies in reducing conditions (D) on tibialis anterior (TA) muscle lysates from control (Ctrl) or C26-bearing mice. In (D), spleen lysate (5 μg) was added as positive control for CD45 expression. (E) Quantification of total CD45 and HMGB1 protein levels normalized on GAPDH. A.U. = arbitrary unit (n ≥ 4 mice/group). (F,G) Western blot probed with anti-HMGB1 antibody in non-reducing conditions on TA muscles isolated from control or C26-bearing mice (F). The upper and lower bands correspond to the fully-reduced HMGB1 (frHMGB1) and the disulphide-HMGB1 (dsHMGB1) isoforms, respectively. (G) Quantification of HMGB1 redox isoforms percentage. A.U. = arbitrary unit (n ≥ 4 mice/group). (H,I) Western blot probed with anti-CD45, anti-HMGB1, and anti-GAPDH antibodies in reducing conditions on C26 cultured cells (Cells) and on tumoral masses (Tumors) isolated from mice injected with C26 cells (H). Quantification of total CD45 and HMGB1 protein expression normalized on GAPDH (I). A.U. = arbitrary unit (n = 4 cell replicates and n = 5 mice for tumoral masses). (J,K) Western blot probed with anti-HMGB1 antibody in non-reducing conditions on cultured C26 cells (Cells) and on tumoral masses (Tumors) isolated from C26-bearing mice (J). Quantification of HMGB1 redox isoforms percentage in cultured C26 cells (Cells) and in tumoral masses (Tumors) isolated from C26-injected mice (K; n = 4 cell replicates and n = 5 mice for tumoral masses). Data represent the means ± SE [file Image_2.TIFF]

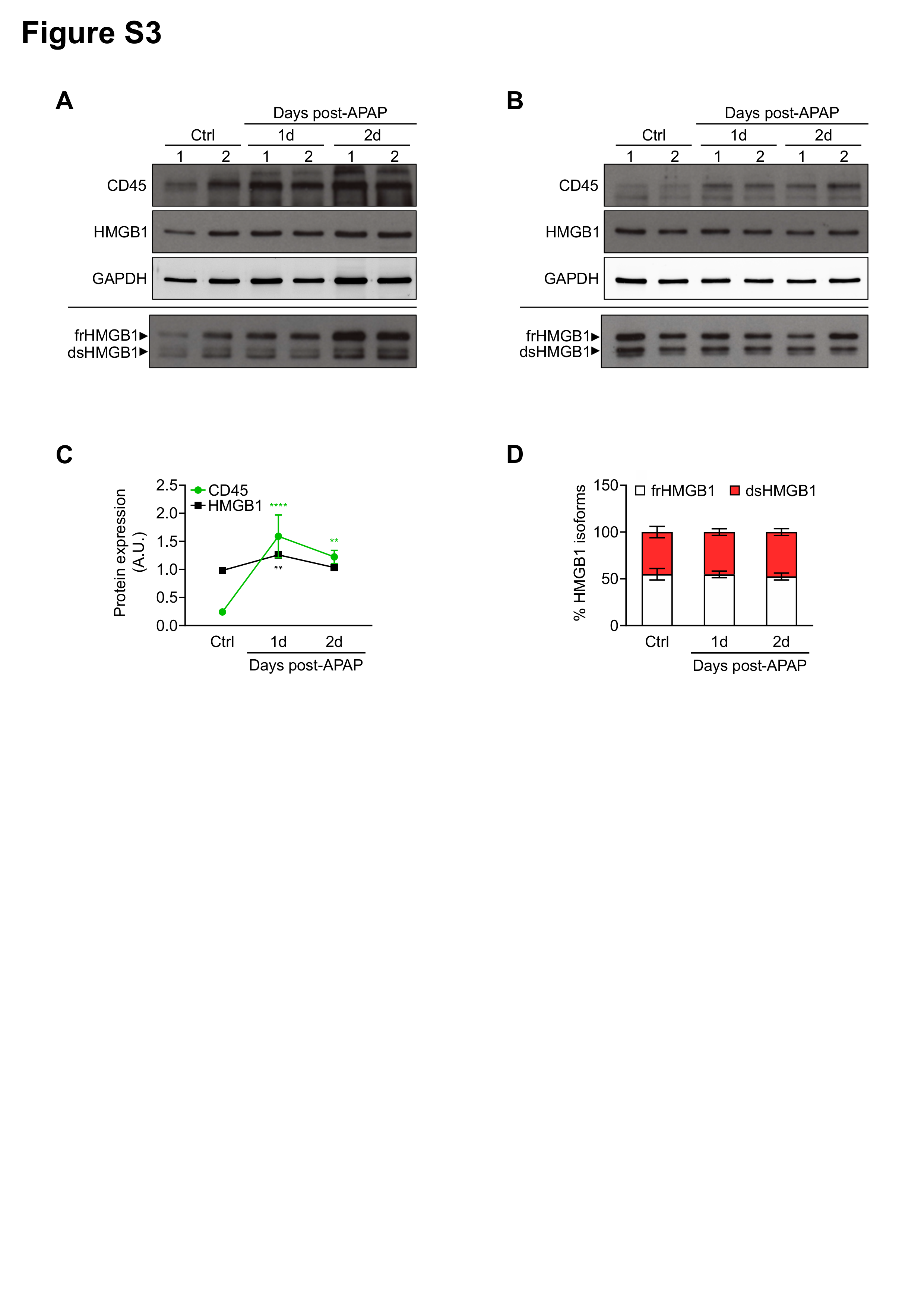

Supplement: Supplementary Figure 3 — CD45 and HMGB1 redox isoforms expression in liver after drug intoxication. Drug-induced liver injury (DILI) was induced by i.p. injection of acetaminophen (APAP), 300 mg/kg (body weight). Liver and intrahepatic leukocytes (IHLs) isolations were performed at indicated time points after APAP treatment. (A) Western blot probed with anti-CD45, anti-HMGB1, and anti-GAPDH antibodies in reducing conditions (upper panels) or probed with anti-HMGB1 antibody in non-reducing conditions (lower panel) on IHLs isolated from control (Ctrl) and APAP-treated mice at indicated time points. In the lower panel, the upper band corresponds to the fully-reduced HMGB1 (frHMGB1) and the lower band to the disulphide-HMGB1 (dsHMGB1). (B) Western blot probed with anti-CD45, anti-HMGB1, and anti-GAPDH antibodies in reducing conditions (upper panels) or probed with anti-HMGB1 antibody in non-reducing conditions (lower panel) on liver lysates of control (Ctrl) and APAP-injected mice at indicated time points. (C) Quantification of total CD45 and HMGB1 protein expression in control (Ctrl) and APAP-treated mice at indicated time points. A.U. = arbitrary unit (n = 4 mice/group). (D) Quantification of HMGB1 redox isoforms percentage in liver lysates from control (Ctrl) and APAP-treated mice (n = 4 mice/group). Data represent the means ± SEM and statistical significance was calculated by One-way (C) and Two-way ANOVA (D). **P < 0.01; ****P < 0.0001. [file Image_3.tiff]
